# Developments in Viral Vector-Based Vaccines

**DOI:** 10.3390/vaccines2030624

**Published:** 2014-07-29

**Authors:** Takehiro Ura, Kenji Okuda, Masaru Shimada

**Affiliations:** Department of Molecular Biodefense Research, Yokohama City University Graduate School of Medicine, Yokohama, Kanagawa Prefecture 236-0004, Japan

**Keywords:** viral vector, vaccine, CTL, MVA, adenovirus

## Abstract

Viral vectors are promising tools for gene therapy and vaccines. Viral vector-based vaccines can enhance immunogenicity without an adjuvant and induce a robust cytotoxic T lymphocyte (CTL) response to eliminate virus-infected cells. During the last several decades, many types of viruses have been developed as vaccine vectors. Each has unique features and parental virus-related risks. In addition, genetically altered vectors have been developed to improve efficacy and safety, reduce administration dose, and enable large-scale manufacturing. To date, both successful and unsuccessful results have been reported in clinical trials. These trials provide important information on factors such as toxicity, administration dose tolerated, and optimized vaccination strategy. This review highlights major viral vectors that are the best candidates for clinical use.

## 1. Introduction 

Viral vectors are regarded as potential tools for gene therapy and vaccines. Their utility is based on the ability of viruses to infect cells. In general, the advantages of viral vectors are as follows: (a) high efficiency gene transduction; (b) highly specific delivery of genes to target cells; and (c) induction of robust immune responses, and increased cellular immunity.

The concept of viral vector vaccines is different from that of subunit vaccines, as the latter help prevent infectious diseases by eliciting a humoral response. Recombinant viral vectors have potential for therapeutic use because they enable intracellular antigen expression and induce a robust cytotoxic T lymphocyte (CTL) response, leading to the elimination of virus-infected cells. Despite their efficacy, viral vectors present unavoidable problems that need to be addressed. In the near future, viral vector-based vaccines may be increasingly used to fight major diseases, such as HIV-1 and malaria. In some vectors, stable expression of the interesting gene is achieved via viral integration mechanisms. Integration into the host genome can lead to cancer. Another obstacle to the clinical use of viral vectors is the presence of pre-existing immunity against the vector. This is caused by previous exposure to the virus and the production of neutralizing antibodies that reduce vaccine efficacy. 

The development of viral vectors requires a high biological safety level in order to gain public acceptance. Therefore, non- (or low-) pathogenic viruses are often selected. In most cases, viruses are genetically engineered to reduce or eliminate pathogenicity. Additionally, most viral vectors are replication-defective. For example, in adenovirus-based vectors, the E1A and E1B encoding regions, which are needed for replication in infected cells, are deleted and replaced with the target gene.

When using a viral vector, it is important to assess the potential implications by understanding the epidemiological and virological characteristics. In this review, we describe several representative viral vectors with respect to risk and benefit, optimized vector-design attempts, and clinical results. Moreover, we discuss the combined use of viral vectors in prime-boost vaccination regimens. 

## 2. Summary of Viral Vectors

The concept of the viral vector was introduced in 1972. Jackson *et al.* created recombinant DNA from the SV40 virus by genetic engineering [1]. Subsequently, Moss *et al.* reported the use of vaccinia virus as a transient gene expression vector in 1982 [2,3]. Several types of viral vectors have been developed, and they have been used in animal studies and clinical trials.

The specific properties of a vector are determined by the virus from which it derives. Each vector has distinct advantages and disadvantages (Table 1). Vaccinia virus and adenovirus are the most widely used vectors because they can induce a robust immune response, specifically involving CTL, against the expressed foreign antigens. Generally, viral vectors achieve high immunogenicity without an adjuvant. Viral components stimulate the innate immune response, leading to the production of interferons and inflammatory cytokines [4].

Viral vector-based vaccines require assessment of efficacy and safety, including immunogenicity, genetic stability, ability to evade pre-existing immunity, replication deficiency or attenuation, and genotoxicity. Additionally, the cost-effectiveness must also be evaluated because infectious diseases are a problem in developing countries. Thus, it is necessary to consider the large-scale manufacturing of viral vectors. Typically, the manufacturing of viral vectors involves propagation in suitable cell lines.

The European Medicines Agency (EMA) recently provided guidelines on quality for non-clinical and clinical aspects [5]. These guidelines offer useful recommendations for the effective administration of live, recombinant viral vector-based infectious vaccines.

**Table 1:** Advantages and disadvantages of major viral vectors.

Virus	Advantages	Disadvantages	Major Clinical/Preclinical Studies
Retrovirus	Long-term gene expression	Generation of replication-competent virus Potential for tumorigenesis Infects dividing cells only	[6]
Lentivirus	Long-term gene expression Infects non-dividing and dividing cells	Generation of replication-competent virus Potential for tumorigenesis	[7,8]
Vaccinia virus	High immunogenicity Safety: used as a smallpox vaccine High titer production	Pre-existing immunity	[9]
Adenovirus	High immunogenicity Safety: used in many clinic trails High titer production	Pre-existing immunity	[10]
Adeno-associated virus	Long-term gene expression Non-pathogenic virus	Low titer production	[11]
Cytomegalovirus	Induces a unique CTL response Protects against SIV infection in an animal model	Pre-existing immunity Risk of pathogenesis in specific individuals	[12]
Sendai virus	High immunogenicity	Pre-existing immunity	[13]

## 3. Viral Vectors

### 3.1. Poxviruses as Vaccine-vectors

Vaccinia virus, a member of the poxvirus family, is a large, complex, enveloped virus. The virus is traditionally used for the smallpox vaccine, and its efficacy and safety have been demonstrated. During vaccine development, highly attenuated vaccinia virus strains have been generated, including replication-competent (LC16m8) and replication-deficient (NYVAC, ALVAC, TROVAC and MVA) strains. These strains are most frequently used for vaccinia virus vector production, although numerous strains of vaccinia virus exist in humans and animals.

Modified vaccinia Ankara (MVA) is a highly attenuated strain derived from the vaccinia strain Ankara. MVA has lost the 15% of the vaccinia genome and the ability to replicate in mammalian cells. MVA has been safely administered to over 120,000 individuals as a smallpox vaccine [14]. For priming, 1 × 10^6^ infectious units (IU)/dose of MVA was administered. LC16m8 is derived from the Lister strain, which has a narrow host range for replication. LC16m8 has been evaluated in 50,000 children without severe side effects [15]. NYVAC is generated from the Copenhagen strain by genetic deletion. ALVAC and TROVAC are derived from the canarypox and fowlpox viruses, respectively. 

Vaccinia virus has a linear, double-stranded DNA genome approximately 190 kb in length. Because of its large size, the viral genome has a high capacity for foreign gene insertion. Indeed, the vector accepts approximately 25 kb of foreign genetic material.

The first vaccinia virus-based gene expression vector was described in 1982 [2,3]. Since then, many studies have shown that vaccinia virus vector-based vaccines can induce a robust immune response against foreign antigens because of high transgene expression [16,17]. In additional, the vectors activate a strong innate immune response mediated by Toll-like receptors (TLRs) and the inflammasome, resulting in an adjuvant effect [18,19].

Currently, many clinical trials are evaluating the use of vaccinia virus vectors. Various diseases are targets for vaccines, such as HIV-1 [20,21,22], hepatitis [23], influenza [24], malaria [25,26], tuberculosis (TB) [27], and cancer [28]. Most of the vaccines are intended to induce a robust CTL response against foreign antigen. In clinical trials, vaccinia virus vectors were well tolerated, although severe adverse events were observed in some studies when the MVA vector was administered at a dose over 10^8^ pfu [29]. In 2009, a Phase III ALVAC-based HIV-1 vaccine demonstrated a modest protective effect. This trial was the first to provide evidence for the efficacy of an HIV-1 vaccine in a large-scale study [9].

Another advantage of vaccinia virus is that a large-scale manufacturing method was established for the production of the smallpox vaccine. For example, Bavarian Nordic A/S produced 20 million doses of smallpox vaccine (named IMVAMUNE^®^), which were delivered to the U.S. government for emergency use [30]. For large-scale manufacture, the MVA vector is generally produced in chick embryo fibroblast (CEF) cells. Specifically, the plasmid with the recombinant transgene is transfected into CEF cells using vaccinia virus. The recombinant vectors that propagate in the CEF cells are purified by centrifugation. Recently, AGE1.CR [31] and EB66 [32] cell lines have been developed for improved MVA production. 

A presumed disadvantage of vaccinia virus vectors is the limited immunogenicity observed in smallpox-vaccinated individuals. Pre-existing immunity against the poxvirus may reduce the vaccine’s efficacy [33]. According to some clinical trials, the effect of pre-existing immunity is moderate [34,35].

Today, efforts to improve vaccinia virus vectors continue. To improve immunogenicity, the development of stronger promoters and the removal of immunomodulatory MVA genes have been reported [36,37]. 

Ultimately, vaccinia virus vectors have the potential to be employed as highly immunogenic and well-tolerated vaccines. Their use has historical precedent in the smallpox vaccine, and a manufacturing process has been established. 

### 3.2. Adenovirus Vectors

Approximately 50 human adenovirus (Ad) serotypes have been identified. Among them, human Ad serotype 5 (Ad5) has been widely investigated as a gene delivery vector because it can be easily produced in high titers. Ad5 contains a 36-kb double-stranded, linear DNA genome; it has a capsid shell with hexon and penton structures and no envelope. Because the Ad fiber protein mediates Ad binding to the coxsackievirus and Ad receptor (CAR), it is a major determinant of viral tropism. Various clinical syndromes are causes by Ad infection, including acute respiratory disease, pharyngoconjunctival fever, and gastroenteritis.

Recombinant Ad vectors are widely used because of their high transduction efficiency, high level of transgene expression, and broad range of viral tropism. They can infect both dividing and non-dividing cells. 

Most Ad vectors are replication-defective because of deletion of the *E1A* and *E1B* viral gene region. Often, the *E3* genes are also deleted to provide space for the transgene. A popular method for Ad vector production involves two steps. First, the Ad vector plasmid is transfected into E1-complementing cell lines (the 293 cell lines are frequently used). The Ad vector then propagates in the infected 293 cells in culture. Second, the vector is collected from infected cells and purified by ultracentrifugation.

Because most people have been exposed to an Ad serotype, the presence of pre-existing anti-Ad immunity is a disadvantage of the Ad vector. Ad contains three main structural proteins, hexon, penton, and fiber. These proteins are the major targets of the humoral and cellular immune responses against Ad5 [38,39]. Antibodies against the hypervariable regions (HVRs) of the hexon protein dominate the neutralizing responses [40]. Modification of these HVRs and the fiber knob domain has been investigated as a way to evade pre-existing immunity [41,42,43].

Although replication-defective Ad has reduced its overall virulence, a further improvement in the clinical safety profile of Ad vectors is required. For example, because CAR is expressed at a high level in hepatocytes, Ad5-based vectors have a strong tropism for liver parenchymal cells, which increases hepatotoxicity [44]. Modification of the fiber protein of Ad can alter its tropism and reduce liver toxicity [45]. Another approach is modulation of the host immune response by reducing viral gene expression. Deletion of the E2 and E4 viral gene regions lowers toxicity by reducing the vector-derived immune response in infected cells. This vector is called a 2nd generation Ad vector [46].

Modification of the Ad fiber protein also improves the efficiency of gene transduction because fiber protein determines the virus’s tropism. The RGD-fiber Ad vector, which incorporates an Arg-Gly-Asp (RGD) peptide into the fiber knob domain, enhances transduction into T cells and dendritic cells [47]. Our group generated a highly immunogenic chimeric Ad5 vector that expresses the Ad35 fiber protein [45].

Recombinant Ad vector-based vaccines have been examined in clinical trials against HIV-1 [48,49], influenza [50], and solid tumors [51]. Ad vectors are well tolerated, and a Phase I study of an HIV-1 vaccine identified 10^10^ virus particles (VP) as the best-tolerated dose [52]. Despite these efforts, a major Phase IIb trial failed, as the Ad vector further amplified a pre-existing immune response against Ad5, during a STEP Study [10]. This result revealed that vector-based vaccines such as the above could provide conducive environments for HIV-1 replication, thereby contradicting the clinical safety outcomes of a previous HIV-1 study [53]. On the contrary, a Phase I Ad5-based TB vaccine demonstrated immunogenic efficacy, in spite of a pre-existing anti-Ad immunity [54]. In order to evaluate the safe use of Ad-based vectors, a better understanding of the host immune response against different antigens seems necessary.

### 3.3. Adeno-Associated Virus Vectors

Adeno-associated virus (AAV) is a small, single-stranded DNA virus that lacks an envelope. AAV is a nonpathogenic viral vector; the virus has low immunogenicity and has never shown any pathogenicity. The AAV genome integrates into the human genome at a specific site on chromosome 19q. The integration involves the *Rep* region and inverted terminal repeats (ITR) at both ends of the viral genome. Thus, AAV vectors provide long-term transgene expression. The virus can infect both dividing and non-dividing cells and has broad tropism for many different cell types. 

Twelve AAV serotypes in humans and more than 100 AAV serotypes in diverse animal species, including nonhuman primates, canines, and fowl, have been found. AAV2 is commonly used as a vector in preclinical and clinical studies. Each serotype has unique receptors, and the tissue specificity is determined by the capsid serotype. For instance, AAV-1 and AAV-7 are best for the transduction of skeletal muscle [55,56]. AAV6 and AAV5 infect airway epithelial cells efficiently [57,58]. The AAV vector also efficiently transfers transgenes into the brain [59], muscle [60], lung [61], gut [62], liver [63], and eye [64].

Generally, recombinant AAV vectors are generated by deletion of the *Rep* and *Cap* coding regions between the ITRs. These regions are used for endogenous transgene expression. Owing to the deletion of these regions, AAV vectors cannot integrate into the host genome, and their DNA also persists in an episomal form. This preferable feature in the AAV vectors boosts their safety profile, by preventing the onset of tumorigenesis. The AAV vector has a transgene capacity of approximately 4.5 kb, which is lower than other viral vectors, including herpes virus (30 kb), adenovirus (8–10 kb), and retrovirus (7–8 kb).

During manufacturing, AAV requires a helper virus to replicate. Thus, AAV vector production requires complementation of adenovirus regions VA, E1, E2, and E4. These proteins are provided either by plasmid co-transfection or by adenovirus infection of host cells. Compared to other viral vectors, the AAV vector has low-titer production efficiency. Therefore, high-efficacy and large-scale production methods have been developed. For example, the use of baculovirus system enhances AAV vector production [65]. Baculovirus systems easily provide helper functions for vector production and insect cell growth [66].

An alternative way to compensate for low-titer production is to reduce the administration dose. In human clinical trials, a dose of 10^12^–10^13^ genome copies/kg body weight of AAV is commonly administered. To reduce the dose, it is necessary to improve the transduction efficiency and increase the immunogenicity of the vector. To enhance AAV immunogenicity, capsid modification vectors have been developed. AAV comprises three structural capsid proteins, VP1, VP2, and VP3. Generating of mixture capsids from different serotypes can alter tropism and increase the efficiency of gene delivery to target cells or tissues [67].

When using AAV vectors, assessment of the risk of genetic toxicity is important because AAV vector may require host genome integration for viral gene expression. With regard to the tumorigenicity of AAV vectors, conflicting results have been reported in animal studies [68,69]. 

Numerous clinical studies have investigated the use of AAV vectors for gene therapy in the treatment of Parkinson’s disease [70], Alzheimer’s disease [71,72], cardiac disease [73], and prostate cancer [74]. In 2012, the EMA approved an AAV1 vector to deliver the lipoprotein lipase (LPL) gene in patients with LPL deficiency [11]. Many animal studies have examined vaccine vectors against HIV-1 [75,76,77], influenza [78], and papillomavirus [79]. These animal studies indicate a potential for AAV-based vaccines. However, human clinical trials are rare because AAV vectors induce weak humoral and cellular immunity compared to other viral vectors (e.g., Ad, MVA). Moreover, infectious vaccines normally target a large population, healthy people, and a wide range of ages, including children and adolescents. Thus, AAV vector-based vaccines require stronger safety measures and greater cost-effectiveness than AAV-based gene therapies.

### 3.4. Retrovirus Vectors

A retrovirus is an enveloped, single-stranded RNA virus that contains reverse transcriptase. Retrovirus vectors are typically replication-defective, and most are of murine or avian origin. Among them, the Moloney murine leukemia virus (MoMLV) has been widely investigated. Retroviruses require genome integration for gene expression. Thus, retrovirus vectors similar with AAV, provide long-term gene expression. The genome size is approximately 7–11 kb and the vector can easily harbor 7–8 kb long foreign DNA inserts. Retroviruses display low immunogenicity and most patients do not show any pre-existing immunity to retroviral vectors. However, retroviruses are associated with various diseases. For example, MoMLV causes leukemia and lymphoma, although it is species-specific.

In clinical studies, retroviral vector-based gene therapy has been implemented in patients with an X-linked severe combined immunodeficiency (SCID-X1) or malignant glioma [6,80]. The therapy for SCID-X1 demonstrated high efficacy. However, four out of the 10 treated patients eventually developed lymphoma [81]. This safety issue also arose during preclinical trials [82,83]. The onset of tumorigenesis resulted from the integration of viral LTRs into proto-oncogenes. As retroviral vectors preferentially integrate near cellular gene promoters that regulate cell replication, non-integrating and self-inactivating (SIN) vectors could potentially reduce the risk of tumorigenesis [84,85]. SIN vectors contain partially deleted LTRs and are rendered inactive during vector production. Owing to this reason, SIN vectors have been recommended for clinical trials targeting SCID-X1 [86].

### 3.5. Lentivirus Vectors

Lentiviruses constitute a subclass of retroviruses. Lentiviruses infect both non-dividing and dividing cells, whereas retroviruses only infect dividing cells. Thus, lentiviruses generally exhibit broader tropism than retroviruses. Several proteins such as tat and rev regulate the replication of lentiviruses. These regulatory proteins are absent in retroviruses. HIV is a well-known lentivirus that has been engineered into a transgene delivery vector. The advantages of lentiviral vector are similar to those of retroviral vectors. Although lentiviruses can potentially trigger tumorigenesis, the risk is lower than that of retroviral vectors, as the integration sites of lentiviruses are away from the sites harboring cellular promoters.

As of now, several types of HIV-based vectors have been generated, by deleting the HIV viral envelope and some of the regulatory genes not required during vector production. Instead of parental envelope, several chimeric or modified envelope vectors are generated because it determines the cell and tissue specificity. For example, VSV/HIV-1-based vectors contain the envelope glycoprotein derived from the vesicular stomatitis virus (VSV), and are used during vaccination owing to the broad range tropism exhibited by VSV [87]. Envelopes derived from filovirus [88], MoMLV [89] and measles [90] have also been used in designing therapeutic vectors. An HIV-1 based vector called VRX496 (developed by VIRxSYS Corporation) was used for HIV-1 gene therapy during recent clinical trials. These trials demonstrated a favorable safety profile and a potential application in gene therapy [7,8].

The virulence of HIV-1 raises serious concerns. Feline immunodeficiency virus (FIV)-based vectors have hence been developed, as the virulence of lentiviruses is highly species-specific [91]. FIV vector vaccines developed to combat HIV-1 and the Herpes simplex virus have demonstrated significant potential [92,93].

### 3.6. Cytomegalovirus Vectors

Cytomegalovirus (CMV) is a member of the herpesviruses. Several species-specific CMVs have been identified. Among them, human CMV (HCMV), also known as human herpesvirus type 5, has been most thoroughly investigated. HCMV contains a 235-kb double-stranded linear DNA genome, which is surrounded by a capsid. The envelope contains the glycoproteins gB and gH, which bind to cellular receptors. CMV often goes unnoticed because its pathogenicity is mild in people who are immunologically healthy. Pathogenesis is a risk only in pregnant woman and immunocompromised individuals. 

Hansen *et al.* reported that a rhesus CMV (RhCMV) vector-based vaccine protected against SIV infection and eliminated SIV [12]. Interestingly, the RhCMV-based vaccine elicits a unique MHC class II-restricted CTL response that recognizes a broad range of antigen epitopes. Such features have not been observed with MVA- or Ad5-based vaccines [94]. This HCMV vector-based vaccine has been recently expected to one of the best effective vaccine against HIV infection.

### 3.7. Sendai Virus Vectors

Sendai virus (SeV) is an enveloped, single-stranded RNA virus of the family Paramyxovirus. It is also known as murine parainfluenza virus type 1 or hemagglutinating virus of Japan. SeV causes bronchopneumonia in mice, but is considered non-pathogenic in humans. However, as SeV is highly homologous to the human parainfluenza type 1 (hPIV-1) virus, a pre-existing immunity against hPIV-1 also works against SeV. Hara *et al.* showed that SeV-specific neutralizing antibodies are detected in a majority of adult people [95]. The SeV genome encodes six protein and two envelope glycoproteins, HN and F proteins, that mediate cell entry. These proteins also determine its tropism. Thus, lack of F protein results in a replication-defective virus and improves the safety of the vector. The SeV vector produced in the packaging cell expresses the F protein. An F gene-deleted and transgene-inserted genome is transfected into the packaging cell. Additionally, SeV contains RNA dependent RNA polymerase and viral genome localizes to the cytoplasm. This ensures that fast gene expression occurs soon after infection and the genotoxic advantage of SeV. The SeV vector exhibits highly efficient gene transfer and transduces both dividing and non-dividing cells. The SeV vector efficiently transduces human airway epithelial cells, and it is often administered by a mucosal (oral and nasal) route. Intranasal administration can potentially reduce the influence of a pre-existing immunity to SeV, as compared to intramuscular administration [96]. Compared to other viral vectors, its transgene capacity (3.4 kb) is low, which limits the use of the SeV vector.

The SeV vector has been used for gene therapy and in a vaccine in human trials [13,97]. A clinical trial of an SeV vector-based HIV-1 vaccine is underway (NCT01705990). The trial is using a replicating SeV vector, rather than a non-replicating vector, in order to elicit a more effective immune response.

## 4. Combination Vaccine Regimens

The prime-boost regimen is a common method for vaccination. As mentioned above, multiple vaccinations with a single viral vector are considered ineffective because of the induction of anti-vector immunity. To overcome this problem, a DNA prime and viral vector boost strategy is often used. Many studies have shown that this strategy induces a protective CTL response. Another approach is the combined use of different types of viral vectors. This approach has the potential to induce a more robust immune response than any other method. As a combination regimen, MVA and Ad vectors are generally used because both vectors have high immunogenicity. These vectors elicit a much higher immune response when using a prime-boost regimen [98,99]. However, our group found that co-administration of Ad and MVA vectors suppressed transgene expression via soluble factors secreted by virus-infected cells. In an *in vitro* experiment, the MVA vector was shown to infect cell culture supernatant suppressed Ad vector transgene expression [100]. 

Another approach is to combine a viral vector with a currently available vaccine. For instance, the BCG vaccine, a live, attenuated vaccine against TB, induces limited protection, especially in adolescents and adults. The ability of an MVA85A boost regimen to enhance protective immunity following BCG vaccination was examined. A Phase IIb trial of MVA85A failed, but the use of viral vectors to enhance a priming immunization remains a possibility [27]. 

## 5. Conclusions

In conclusion, several viral vectors have been used in vaccine production and in gene therapy. In this review, we have described several candidate viral vectors with potential clinical applications. The MVA vector and the Ad vector are the most preferred therapeutic vectors against HIV-1. The CMV vector elicits a unique immune response, whereas the SeV vector can potentially induce mucosal immunity. Viral vector-based vaccines can be easily manufactured alongside traditional vaccines in large manufacturing units, and their safety profiles can be tested easily. The MVA virus, the poliovirus, and the measles virus, have all been investigated for viral vector use.

The first clinical trial of a therapeutic retroviral vector took place in 1990. Subsequent clinical studies have raised serious concerns regarding genotoxicity, mainly due to possible viral genome integration. The AAV vector has the ability to express episomal genes without integrating itself into the host genome, and has hence been approved by the EMA for clinical use.

Today, numerous viral vectors are being investigated. Each vector has unique advantages, as described above. Exploiting their advantages will increase their potential and hasten the clinical use of viral vector-based vaccines. These vaccines can potentially induce a robust immune response in tissues and cells and achieve targeted delivery. Early-phase trials show that they can be tolerated well in humans. Ongoing efforts to design and optimize vaccination regimens will eventually result into the development of new vaccines. 
